# Acute Intestinal Obstruction in a Case of Cerebrovascular Accident

**DOI:** 10.7759/cureus.63010

**Published:** 2024-06-24

**Authors:** Shubhangi Kanitkar, Sai Priya Ande, Kranthi Dandi, Muskaan Ahlawat, Akshata Borle

**Affiliations:** 1 Department of Medicine, Dr. D. Y. Patil Medical College, Hospital & Research Centre, Dr. D. Y. Patil Vidyapeeth (Deemed to be University), Pune, IND; 2 Department of Gastroenterology, Dr. D. Y. Patil Medical College, Hospital & Research Centre, Dr. D. Y. Patil Vidyapeeth (Deemed to be University), Pune, IND

**Keywords:** ogilvie’s syndrome, obstipation, abdominal distension, neostigmine, intestinal pseudo-obstruction

## Abstract

Ogilvie's syndrome represents an acute form of intestinal obstruction that occurs in the absence of a detectable mechanical blockage impeding fecal passage. Hence, it is also given the name of intestinal pseudo-obstruction. It has been deemed a disease of imbalance between the arms of the autonomic nervous system with an increase in parasympathetic outflow. Most often, it has an antecedent surgical or medical illness. There is evidence for the use of IV neostigmine in such cases to prevent imminent intestinal ischemia and perforation. In the case of a non-responder, decompression of the bowel using a colonoscope and surgery have also been tried to relieve the symptoms. In the case that follows, a middle-aged man developed progressive abdominal distension in the course of his recovery from an ischemic cerebrovascular accident. Initially, he received conservative treatment for 48 hours. Subsequently, he was given IV neostigmine, which relieved his symptoms.

## Introduction

Ogilvie's syndrome, often referred to as acute colonic pseudo-obstruction (ACPO), is a disorder marked by significant colonic distension without mechanical obstruction. Ogilvie's syndrome is more likely to occur in patients with antecedent medical and surgical problems. Conservative therapy is often effective in managing Ogilvie's syndrome. If left undiagnosed and unmanaged, Ogilvie's syndrome-related persistent distension can result in perforation, which has a high fatality risk.

The parasympathetic nervous system often increases gut motility, while the sympathetic nervous system decreases it. The etiology of ACPO has been attributed to disorders of the autonomic nervous system, characterized by parasympathetic dysfunction, sympathetic dysfunction, or a mix of both [1]. The effectiveness of neostigmine implies that parasympathetic dysfunction is the underlying reason, which contradicts Ogilvie's original notion of sympathetic deprivation [2].

According to a study [3], 94.5% of the patients had a coexisting surgical or medical condition. Although six previous extensive studies have found a link with orthopedic surgery, infection, heart disease, and recent postoperative care, other variables that contribute to the development of Ogilvie's syndrome include drug use and imbalances in electrolytes [4,5].

## Case presentation

A 48-year-old male with a known case of hypertension presented at a local hospital due to the sudden onset of right-sided weakness and was diagnosed with an acute ischemic cerebrovascular stroke. He required intubation due to a low Glasgow Coma Scale score and was managed conservatively, followed by successful extubation after two days. Three days after extubation, he developed progressive abdominal distension without passing stool or gas, leading to his referral to our institution for further management.

**Figure 1:**
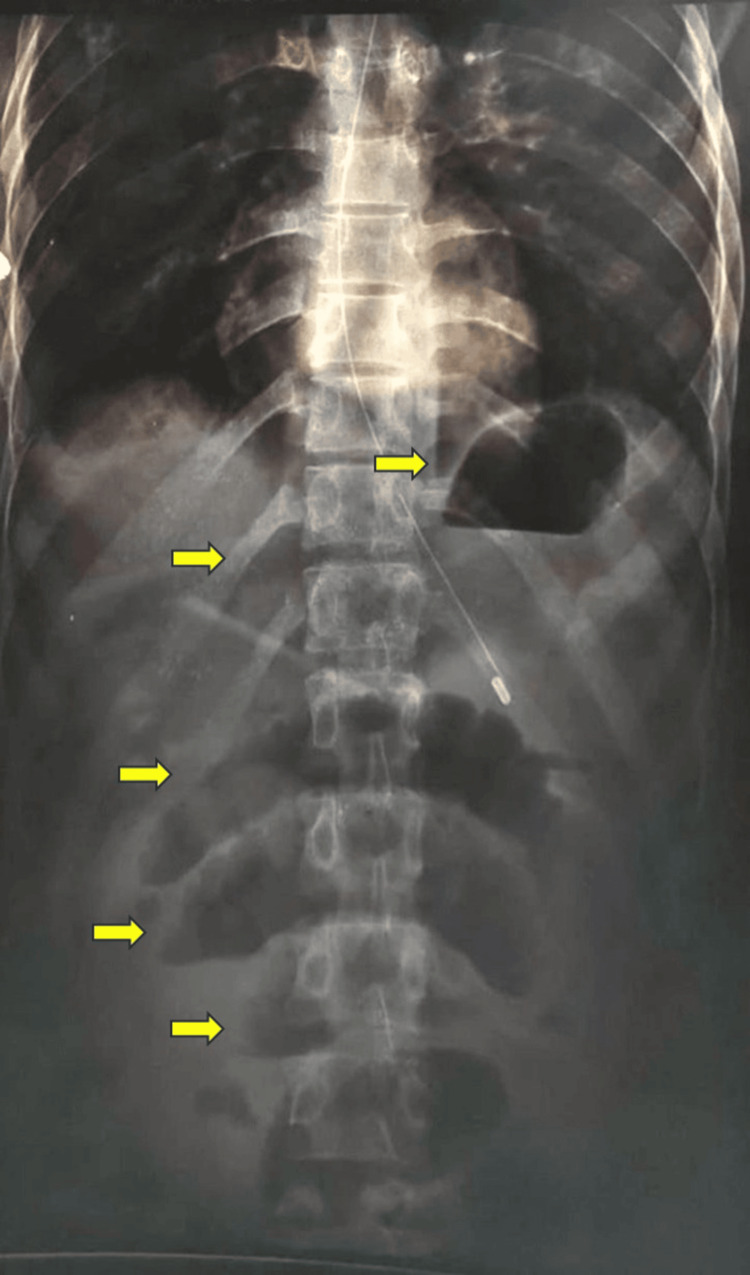
X-ray of the erect abdomen upon the arrival of the patient at the hospital The yellow arrows demonstrate multiple air-fluid levels.

Upon examination, the pulse rate was 104 beats per minute, the blood pressure was 100/70 mmHg, and the oxygen saturation was 98% in room air. An abdominal examination revealed a distended abdomen with a tympanic note on percussion and absent bowel sounds. The neurological assessment indicated a muscle power of grade 3/5 in both the right upper and lower limbs, accompanied by brisk reflexes and a right-sided extensor plantar response. An abdominal roentgenogram displayed dilated bowel loops with multiple air-fluid levels (Figure 1). The patient was not allowed oral feeds, and a nasogastric tube was inserted.

The laboratory tests indicated hypokalemia, which was treated with intravenous potassium (Table 1). The patient initially received conservative treatment with IV fluids, electrolytes, and nasogastric aspiration for 48 hours. However, his abdominal distension did not resolve.

**Table 1:** Laboratory investigations AST: aspartate transaminase; ALT: alanine transaminase; ALP: alkaline phosphatase; g/dL: gram/deciliter; µL: microliter; mg/dL: milligrams/deciliter; mmol/L: millimoles/liter; U/L: units/liter.

Lab Investigation	Patient Value	Reference Range
Hemoglobin	13.7 g/dL	13.2-16.6 g/dL
Total leukocyte count	9,000/µL	4000-10,000/µL
Platelet count	3,77,000/µL	1,50,000-4,10,000/µL
Urea	18 mg/dL	17-49 mg/dL
Creatinine	0.68 mg/dL	0.6-1.35 mg/dL
Sodium	139 mmol/L	136-145 mmol/L
Potassium	2.87 mmol/L	3.50-5.10 mmol/L
Chloride	104 mmol/L	98-107 mmol/L
Calcium	8.9 mg/dL	8.6-10.2 mg/dL
Magnesium	2.1 mg/dL	1.80-2.40 mg/dL
Phosphorus	2.4 mg/dL	2.6-4.7 mg/dL
Total bilirubin	0.45 mg/dL	0.22-1.20 mg/dL
Direct bilirubin	0.2 mg/dL	Up to 0.5 mg/dL
AST	37 U/L	8-43 U/L
ALT	29 U/L	7-45 U/L
ALP	54 U/L	35-104 U/L

**Figure 2:**
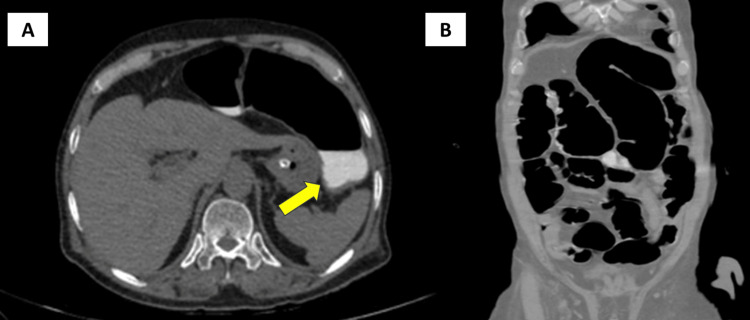
Computerized tomography (CT) of the abdomen and pelvis with contrast (A) The transverse section demonstrates a dilated transverse colon measuring 5.3 cm in its largest diameter with retained positive oral contrast, indicated by a yellow arrow; (B) the coronal section demonstrates dilated large bowel loops with no evidence of a cutoff point and no apparent mechanical obstruction.

**Figure 3:**
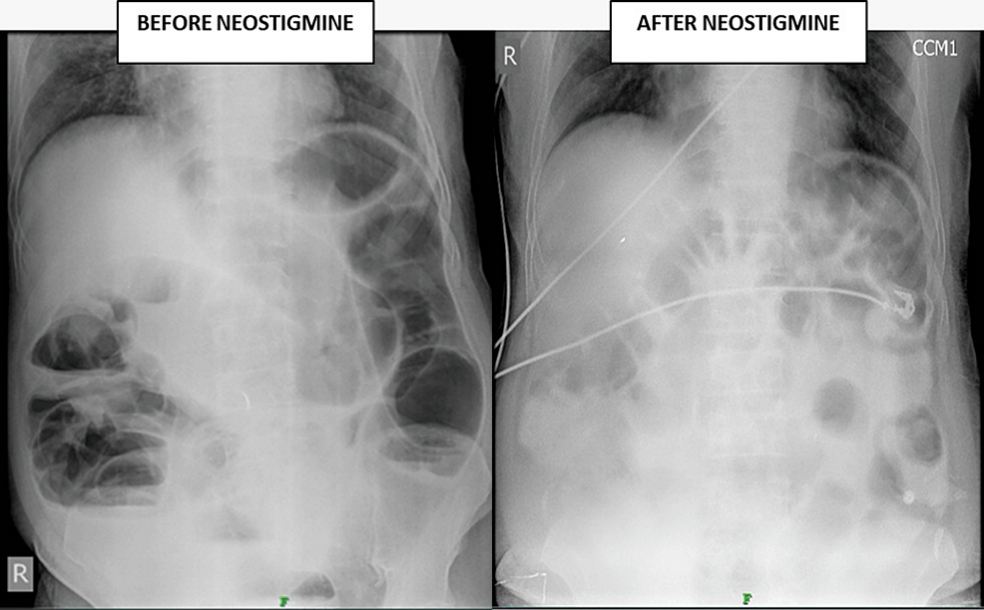
X-ray of the erect abdomen Demonstrates the decrease in air-fluid levels following the administration of injection neostigmine (2 mg IV).

Abdominal and pelvic computerized tomography (CT) imaging showed significantly dilated large bowel loops, measuring up to 5.9 cm in diameter, without evidence of mechanical obstruction (Figure 2). Clinical and radiological findings led to a diagnosis of Ogilvie's syndrome. Despite electrolyte normalization and conservative treatment, the patient's symptoms persisted. Pharmacological management with IV neostigmine was considered as the patient did not have any contraindications to the use of the neostigmine and failed to respond to conservative management. The patient received a 2 mg IV injection of neostigmine over 10 minutes, with atropine available bedside. Continuous clinical and ECG monitoring was conducted for 30 minutes post-injection. The patient did not experience any side effects of the drug, such as bradycardia, hypotension, or hypersensitivity reactions. However, the patient experienced symptomatic relief, as evidenced by the passage of gas and increased gastric secretions via the nasogastric tube (Figure 3). Following the injection of neostigmine, the patient was started on 29.5 g of polyethylene glycol (PEG) syrup for seven days to prevent a recurrence of the condition. Since there was no recurrence of symptoms, the patient was observed for 72 hours and then discharged.

## Discussion

It could be challenging to distinguish mechanical intestinal obstruction from Ogilvie's condition. Patients in both mechanical and pseudo-obstruction groups have symptoms such as stomach pain, nausea, and vomiting. Following neurological illnesses, Ogilvie's syndrome was observed in 9.3% of the cases [5].

Radiographic examinations are essential to differentiate Ogilvie's syndrome from other potential causes of large bowel distension. A cecal diameter of <12 cm is considered safe, while a cecal diameter of >14 cm is associated with an increased risk of perforation [5]. A massively dilated colon is shown on flat and upright radiography, usually confined to the right colon and cecum [5-7]. To rule out mechanical obstruction, a water-soluble contrast enema should preferably be administered. The water-soluble contrast enema may be effective in decompressing ACPO in addition to being diagnostic.

The majority of pharmacologic treatment has been focused on reversing the Ogilvie-related sympathetic-parasympathetic imbalance. For neostigmine, the strongest evidence is available to support medical treatment. Neostigmine is an anticholinesterase that increases synaptic acetylcholine levels by inhibiting acetylcholinesterase. The presence of asthma, chronic obstructive pulmonary disease, use of beta blockers, and recent myocardial infarction are some of the absolute contraindications for the use of neostigmine. Neostigmine was first used in clinical trials in combination with guanethidine. For 48 hours, the patients in this research received conservative therapy. After completing the conservative trial, the patients were administered neostigmine, a parasympathomimetic agent, subsequent to an initial dose of guanethidine, an adrenergic blocker. Clinical improvement was seen in eight out of eleven patients, and none of these patients experienced recurrences, which was similar to the findings in our case discussion [7].

The researchers observed that neostigmine injection resulted in improvement following therapy, providing additional evidence to support the idea that parasympathetic suppression, rather than sympathetic overactivity, causes pseudo-obstruction [8]. Prospective investigations later confirmed neostigmine's effectiveness in curing ACPO [9-13]. With the exception of sporadic, moderate side effects, including perspiration and momentary bradycardia, patients' symptoms improved. The frequency of recurrences ranged from 0% to 33% [7-13]. In the present case, the patient did not experience any bradycardia. Alternative drugs tried in this condition include erythromycin, cisapride, domperidone, and metoclopramide. However, their efficacy has not been extensively evaluated, as in the case of neostigmine.

In case of treatment failure with neostigmine, the other feasible options are endoscopic decompression and surgery. In fact, endoscopic decompression has been deemed to be superior to neostigmine [14]. However, the risk of intestinal perforation by endoscopic methods has to be weighed against the spontaneous perforation that could happen in pseudo-obstruction.

The case in question was identified early, averting the potentially deadly complications of intestinal ischemia and perforation. It is vital for both physicians and surgeons to understand the pathogenesis and the pharmacological and non-pharmacological management of this condition.

## Conclusions

Ogilvie's syndrome is a treatable condition that requires a high degree of clinical suspicion in postoperative patients and those recovering from medical conditions. Prompt diagnosis is crucial for managing patients with pharmacological and surgical interventions if there is no response to conservative management, as illustrated in the abovementioned case. Lack of monitoring for patient response to treatment can lead to potentially fatal outcomes, such as intestinal perforation.

## References

[1] Wells CI, O'Grady G, Bissett IP (2017). Acute colonic pseudo-obstruction: a systematic review of aetiology and mechanisms. World J Gastroenterol.

[2] Batke M, Cappell MS (2008). Adynamic ileus and acute colonic pseudo-obstruction. Med Clin North Am.

[3] Maloney N, Vargas HD (2005). Acute intestinal pseudo-obstruction (Ogilvie's syndrome). Clin Colon Rectal Surg.

[4] Tenofsky PL, Beamer L, Smith RS (2000). Ogilvie syndrome as a postoperative complication. Arch Surg.

[5] Vanek VW, Al-Salti M (1986). Acute pseudo-obstruction of the colon (Ogilvie's syndrome): an analysis of 400 cases. Dis Colon Rectum.

[6] Moons V, Coremans G, Tack J (2003). An update on acute colonic pseudo-obstruction (Ogilvie’s syndrome). Acta Gastroenterol Belg.

[7] Delgado-Aros S, Camilleri M (2003). Pseudo-obstruction in the critically ill. Best Pract Res Clin Gastroenterol.

[8] Hutchinson R, Griffiths C (1992). Acute colonic pseudo-obstruction: a pharmacological approach. Ann R Coll Surg Engl.

[9] Amaro R, Rogers AI (2000). Neostigmine infusion: new standard of care for acute colonic pseudo-obstruction?. Am J Gastroenterol.

[10] Trevisani GT, Hyman NH, Church JM (2000). Neostigmine: safe and effective treatment for acute colonic pseudo-obstruction. Dis Colon Rectum.

[11] Loftus CG, Harewood GC, Baron TH (2002). Assessment of predictors of response to neostigmine for acute colonic pseudo-obstruction. Am J Gastroenterol.

[12] Stephenson BM, Morgan AR, Salaman JR (1995). Ogilvie's syndrome: a new approach to an old problem. Dis Colon Rectum.

[13] Turégano-Fuentes F, Muñoz-Jiménez F, Del Valle-Hernández E (1997). Early resolution of Ogilvie's syndrome with intravenous neostigmine: a simple, effective treatment. Dis Colon Rectum.

[14] Tsirline VB, Zemlyak AY, Avery MJ, Colavita PD, Christmas AB, Heniford BT, Sing RF (2012). Colonoscopy is superior to neostigmine in the treatment of Ogilvie's syndrome. Am J Surg.

